# Molecularly Imprinted Polymeric Nanoparticles as Drug Delivery System for Tenofovir, an Acyclic Nucleoside Phosphonate Antiviral

**DOI:** 10.3390/pharmaceutics16070965

**Published:** 2024-07-21

**Authors:** Thomas Mathieu, Patrick Favetta, Luigi A. Agrofoglio

**Affiliations:** Institute of Organic and Analytical Chemistry (ICOA UMR 7311), University of Orleans, Centre National de la Recherche Scientifique, F-45067 Orléans, France; thomas.mathieu@univ-orleans.fr

**Keywords:** molecularly imprinted polymers (MIPs), submicron polymeric particles, functionalized monomers, acyclic nucleoside phosphonate, controlled release, precipitation polymerization, tenofovir, anti-HIV and -HBV drug, adsorption isotherms, release kinetics

## Abstract

A molecularly imprinted polymer of Tenofovir (1), an FDA-approved acyclic nucleoside phosphonate with antiviral activity, was synthesized using a non-covalent approach. A pre-polymerization complex was formed between (1) and DMAEMA and in-house synthetic *N1*-[(2-methacryloyloxy)ethyl] thymine, with EGDMA as a cross-linker in an MeCN/H_2_O (9:1, 1:1) mixture as a porogen, giving an imprinting factor (IF) of 5.5 at 2.10^−5^ mol/L. Binding parameters were determined by the Freundlich–Langmuir model, Q_max_ and K_a_, and well as the particle morphology for MIP and NIP. Finally, the release profiles, for MIP and NIP, were obtained at 25 °C and 37 °C, which is body temperature, in a phosphate buffer saline, pH 7.4, mimicking the blood pH value, to determine the potential sustained release of our polymeric materials.

## 1. Introduction

**Figure 1:**
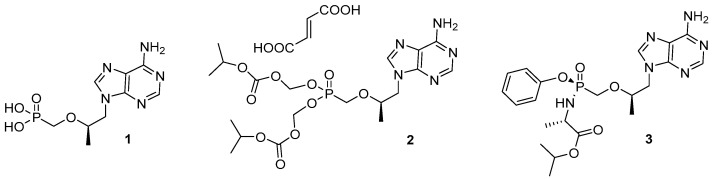
Structures of Tenofovir (1), Tenofovir disoproxil fumarate (2), and Tenofovir alafenamide (3).

Tenofovir (1) ((R)-9-(2-phosphonomethoxypropyl)adenine, PMPA) belongs to the family of acyclic nucleoside phosphonates pioneered by A. Holý and E. De Clercq in 1986 [1]. It is an analogue of adenosine monophosphate, with antiviral activity [2]. As with other nucleosides and nucleotide analogs, in order to be active, Tenofovir must be converted by two subsequent enzymatic phosphorylation processes [3] to Tenofovir diphosphate (analogue of ATP). It then acts as an obligate chain terminator against HIV reverse transcriptase and HBV DNA polymerase. Its poor solubility (2.5 mg/mL) in PBS (pH 7.4) and the negative charge at the phosphonic moiety of Tenofovir hampers its cellular penetration; thus, Tenofovir is converted into two oral formulations through water-soluble prodrugs such as Tenofovir disoproxil fumarate (2) (TDF) [4] and Tenofovir alafenamide (3) (TAF) (Figure 1) [5]. After oral absorption, TDF is quickly converted to Tenofovir in the plasma, then to Tenofovir diphosphate inside cells. The renal toxicity of Tenofovir disoproxil fumarate (TDF) [6,7] primarily arises from the high levels of Tenofovir in the plasma, which lead to accumulation in renal proximal tubule cells. This accumulation can cause mitochondrial toxicity and damage to these cells, impairing renal function. In contrast, TAF remains stable in the plasma, converting to Tenofovir only intracellularly, resulting in lower plasma Tenofovir levels and a different safety profile [8,9,10].

Besides prodrugs, other drug formulations include advanced delivery systems like nanoformulations [11], organic nanoparticles [12], and liposomes [13], which enhance stability, target specific tissues, and improve therapeutic efficacy. To address the limitations of current formulations, a new Tenofovir formulation aims to reduce renal toxicity, decrease bone density loss [14], and improve overall safety [8].

Few drug delivery systems (DDSs) for the release of Tenofovir have been investigated so far. We can quote the work of Spinks et al. [15] and Zidan et al. [16] on liposomal formulations of Tenofovir to enhance its bioavailability. Garcia et al. [17] developed nanodiscoidal bicelles in order to allow Tenofovir to cross the blood–brain barrier to fight persistent HIV-1 infections and neuroinflammation. More recently, Mandal et al. [18] designed a parenteral nanoparticle-based system for the sustained-release of TAF and bictegravir. Finally, Abadi et al. [19] explored the combination of Tenofovir with gold nanoparticles. Recently, a new DDS was developed by Hobson et al. for Tenofovir disoproxil fumarate. This drug is formulated as a novel nanoprecipitation system for long-acting depot injections in order to fine-tune control of the blood concentration of antivirals, to manage HIV infection in humans [20].

Among other DDSs, polymeric materials such as molecularly imprinted polymers (MIPs) have emerged as highly promising DDSs due to their versatility, capacity to encapsulate diverse drugs, excellent stability, and potential for administration through various routes [21,22,23,24]. Originally, MIPs were created as artificial enzymes by Wulff et al. [25]. Then, these materials were used to selectively extract and separate a compound of interest in the presence of hundreds of others in charged matrices, and this new use was initiated by Mosbach’s group [26]. All the teams that have studied this type of material have demonstrated that they are simple, fast, economical, and robust [27,28]. Of course, the synthesis of these polymeric particles presents some challenges. Key considerations include the selection of the template, which is the molecule that creates the selective cavities within the polymer matrix, the porogen, which is a pure solvent or a mixture of solvents capable of solubilizing all the molecules present before synthesis, and the functional monomer, tailored to the specific application. Monomer selection is based on the template’s physical–chemical properties to ensure stable supramolecular complexes through non-covalent, coordinative, or covalent bonding with the chosen template. Stability of the pre-polymerization complex is achieved via acid–base complementarity, hydrogen bonding, or other weak interactions, and typically using a 1:4 template-to-monomer ratio [29]. All of this makes MIPs highly stable with regard to pH, temperature, solvent, and aqueous media, allowing them to protect the drug “inside cavity” from chemical or enzymatic degradations.

In previous work, we showed that these molecularly imprinted materials were capable of selectively recognizing nucleoside derivatives, such as pseudouridine or ribavirin, or nucleotide derivatives, such as adenosine-5′-monophosphate. Their uses range from detecting urinary biomarkers of colorectal cancer in humans to transporting and releasing a molecule that activates cell regeneration in human skin [30,31,32,33]. Our group reported a MIP hydrogel for the drug delivery of the antiviral ribavirin drug [32]. The hydrogels produced demonstrated biocompatibility with model cells, particularly human lung epithelial BEAS-2B cells, showcasing excellent cell viability and a minimal pro-inflammatory response when incubated with imprinted polymers. Notably, viral tests conducted on Influenza A-infected lung cells reveal that imprinted delivery systems, delivering 1 to 3 µg of antiviral agents, exhibit comparable efficacy to a medium containing 30 µg mL^−1^ of the active agent. This underscores the ability of molecularly imprinted polymers as drug delivery systems to enhance local antiviral concentration and improve delivery, especially for low-bioavailability drugs. Thus, among the drug delivery systems, MIPs have become popular as drug vehicles. Unlike the conventional DDSs, thanks to specific polymer–drug interactions, MIPs showed high loading capacity, very high affinity with the drug, and were able to energetically keep a compound within its cavity. The consequences were a sustained release profile of the drug and a minimization of rapid active compound loss, named a burst. As a result, lower doses are required, decreasing vulnerability to adverse reactions and improving safety profiles [34].

**Figure 2:**
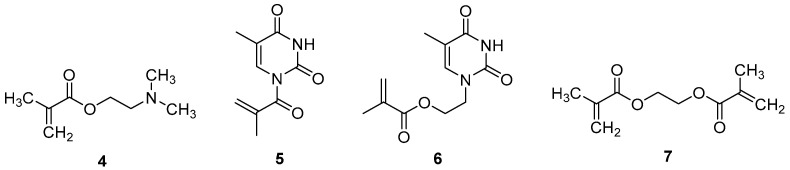
Chemical structures of functional monomers examined in this study and cross-linker: 2-(dimethylamino)ethyl methacrylate (4), *N1*-methacryloyl thymine (5), *N1*-[(2-methacryloyloxy)ethyl] thymine (6), and EGDMA (7).

From this perspective, we decided to translate this know-how to a family of antivirals known as acyclic nucleoside phosphonates, of which Tenofovir (1) is one of the best known, as it is used in the treatment of hepatitis B and AIDS. Thus, as part of our drug discovery program, we report herein the design and the preparation of new DDS system based on MIP technology for the sustained release of Tenofovir. The MIPs are based on non-covalent approaches in which Tenofovir (1) and selected monomers along with the porogen are pre-mixed and allowed to self-associate prior to polymerization. Based on the structure of Tenofovir, the functional monomers examined in this study were as follows: (1) 2-(dimethylamino)ethyl methacrylate (4) (DMAEMA), which is used in the production of cationic polymers, to favor ion-pairing interactions with the negative charges of the phosphonic acid moiety of Tenofovir, and (2) in-house-synthesized monomers based on a thymine scaffold to favor a Watson–Crick base pairing interaction with the adenine of Tenofovir, e.g., the *N1*-methacryloyl thymine (5) and the *N1*-[(2-methacryloyloxy)ethyl] thymine (6) (Figure 2). Ethylene glycol dimethacrylate (EGDMA) (7) was used as a cross-linker and a mixture of acetonitrile–water (90:10, *v*/*v*) as a solvent for the polymerization. This solvent was used in order to dissolve all the starting materials with no/few interferences during the polymerization process.

The adsorption isotherms were determined for the MIP of Tenofovir and the non-imprinted one, taken as reference, to obtain the number of specific cavities, association constants, and payload of the drug delivery systems. The release profiles, for MIP and NIP, were obtained at 25 °C and 37 °C, which is body temperature, in a phosphate buffer saline, pH 7.4, mimicking the blood pH value, to determine the potential sustained release of our polymeric materials.

## 2. Materials and Methods

### 2.1. Reagents

Tenofovir (1) was purchased from Carbosynt Ltd. Commercially available chemicals were of reagent grade and used as received. DMAEMA (4) and EGDMA (7) were purchased from Sigma-Aldrich and used as received, unless otherwise stated. *N1*-methacryloyl thymine (5) and *N1*-[(2-methacryloyloxy)ethyl] thymine (6) were synthesized in-house according to the procedure outlined below. HPLC-grade solvents, acetonitrile (MeCN) and methanol (MeOH), were obtained from Merck and were used as received. All reactions requiring anhydrous conditions were made with glassware after an oven drying step and under dry and inert gas (Ar or N_2_). The reactions were checked by separation of the mixture on 5 × 10 cm silica gel plates (Kieselgel 60F254, E. Merck) by thin-layer chromatography. Purification of the products was carried out by glass column chromatography using Silica Gel 60 M particles (40–63 µm, E. Merck). The NMR spectra (^1^H and ^13^C) were acquired on Avance DPX 250 or Avance 400 Spectrometers (Bruker) with deuterated solvents as the internal standard. Chemical shifts are expressed in ppm and multiplicities are reported as s, d, t, q, ds, and m, meaning singlet, doublet, triplet, quartet, broad signal, and multiplet, respectively. High-Resolution Mass spectra were obtained on a MaXis Q-TOF mass spectrometer (Bruker).

### 2.2. General Procedures for the Synthesis of N1-methacryloyl thymine (5)

To a suspension of thymine (8) (500 mg, 3.97 mmol, 1 eq.) in dry acetonitrile, N,O-bis(trimethylsilyl)acetamide (BSA) (2.44 mL, 9.92 mmol, 2.5 eq.) was added, and the mixture was stirred until solubilization of pyrimidine base (10 min). The mixture was cooled down to −40 °C and methacryloyl chloride (7.94 mmol, 772 µL, 2 eq.) was added dropwise. After 16 h of stirring, the mixture was concentrated under reduced pressure. The crude was purified by recrystallization in a small amount of acetonitrile to give the desired compound (5) as a white solid (423 mg, 55%).

^1^H NMR (400 MHz, d6-DMSO) δ 11.56 (bs, 1H, H3), 7.72 (d, J = 1.7 Hz, 1H, H6), 5.73 (s, 1H, CH_2_=C-), 5.67 (d, J = 1.8 Hz, 1H, CH_2_=C-), 1.98 (s, 3H,=C-CH_3_), 1.83 (s, 3H, CH_3-thymine_). ^13^C NMR (101 MHz, d6-DMSO) δ 171.3 (C=O), 164.4 (C4), 149.6 (C2), 140.1 (CH_2_=C-CH_3_), 135.6 (C6), 124.0 (CH_2_=C-CH_3_), 111.6 (C5), 18.4 (CH_2_=C-CH_3_), 12.4 (CH_3-thymine_). HRMS-ESI (m/z) [M+H]^+^ calcd for C_9_H_11_N_2_O_3_: 195.0764 found: 195.0763.

### 2.3. General Procedures for the Synthesis of N1-[(2-Methacryloyloxy)ethyl] Thymine (6)

#### 2.3.1. Synthesis of N3-Benzoylthymine (9)

Benzoyl chloride (2.8 mL, 23.82 mmol, 3 eq.) was added to a solution of thymine (1 g, 7.94 mmol, 1 eq.) in a 2/5 dry mixture of pyridine and acetonitrile. The mixture was stirred at room temperature for 24 h. Then, all volatiles were evaporated, and the crude was portioned between dichloromethane and water. The organic layer was recovered and evaporated under reduced pressure and the residue was engaged in the second step without purification: A mixture of aqueous potassium carbonate (0.5 M) and 1,4 dioxane was added and the reaction medium was warmed to 70 °C. After 2 h of stirring (complete conversion), the solution was cooled down to room temperature and glacial acetic acid (AcOH) was added to lower the pH to 5 to form a precipitate. Then, the aqueous mixture was filtered off, and 2 successive washes with cold water and diethyl ether gave the desired N3-benzoylthymine as a white solid (1.78 g, 98%).

^1^H NMR (400 MHz, d6-dimethylsulfoxide (d6-DMSO)) δ 11.56 (bs, 1H, NH), 7.97–7.89 (m, 2H, CH_2-bz_), 7.77 (t, J = 7.4 Hz, 1H, CH_-bz_), 7.60 (t, J = 7.7 Hz, 2H, CH_2-Bz_), 7.53 (s, 1H, H6), 1.82 (s, 3H, CH_3-thymine_). ^13^C NMR (101 MHz, d6-DMSO) δ 170.6 (C=O-_bz_), 164.0 (C4), 150.5 (C2), 139.4 (C6), 135.8 (CH_-bz_), 131.9 (Cq_-bz_), 130.7 (CH_2-bz_), 129.9 (CH_2-bz_), 108.3 (C5), 12.2 (CH_3-thymine_). HRMS-ESI (*m*/*z*) [M + H]^+^ calcd for C_12_H_11_N_2_O_3_ 231.0764 found 231.0764.

#### 2.3.2. Synthesis of N1-(2-hydroxyethyl)-N3-benzoylthymine (10)

2-Bromoethanol (1.23 mL, 17.36 mmol, 4 eq.) was added to a suspension of obtained *N3*-benzoylthymine (1 g, 4.34 mmol, 1 eq.) and K_2_CO_3_ (1.8 g, 13.02 mmol, 3 eq.) in dry dimethylformamide (DMF) at 0 °C. The reaction mixture was stirred at room temperature for 2 days. The resulting precipitate was filtrated off and the filtrate was concentrated under reduced pressure. The residue was diluted with ethyl acetate (EtOAc) and washed with H_2_O and brine and dried over Na_2_SO_3_. The residue was concentrated under vacuum and purified with flash chromatography using petroleum ether (PE)/EtOAc as the eluent (1/1 to 2.5/7.5, *v*/*v*), to give *N1*-(2-hydroxyethyl)-*N3*-benzoylthymine as a white solid (770 mg, 2.8 mmol, 65%).

^1^H NMR (400 MHz, d6-DMSO) δ 7.94 (d, J = 8.0 Hz, 2H, CH_2-Bz_), 7.78 (t, J = 8.0 Hz, 1H, CH_-Bz_), 7.71 (s, 1H, H6), 7.60 (t, J = 8.0 Hz, 2H, CH_2-Bz_), 5.02 (t, J = 5.4 Hz, 1H, OH), 3.78 (t, J = 5.4 Hz, 2H, CH_2_-CH_2_-OH), 3.62 (q, J = 5.1 Hz, 2H, CH_2_-OH), 1.85 (s, 3H, CH_3-thymine_). ^13^C NMR (101 MHz, d6-DMSO) δ 169.8 (C=O-_bz_), 163.0 (C4), 149.5 (C2), 143.4 (C6), 135.3 (CH_-Bz_), 131.3 (Cq_-bz_), 130.2 (CH_2-Bz_), 129.4 (CH_2-Bz_), 107.7 (C5), 58.4 (CH_2_-OH), 50.5 (CH_2_-CH_2_-OH), 11.8(CH_3-thymine_). HRMS (ESI): m/z [M+H]^+^ calcd for C_14_H_14_N_2_O_4_: 275.1027 found 275.1026.

#### 2.3.3. Synthesis of N1-(2-hydroxyethyl) thymine (11)

A mixture of *N1*-(2-hydroxyethyl)-*N3*-benzoylthymine (10) (548 mg, 2 mmol, 1 eq.) and 16% (7 N) ammonia in methanol was stirred at room temperature for 4 days. The mixture was concentrated in a vacuum and the resulting residue was purified with flash chromatography using dichloromethane (DCM)/methanol (MeOH) as the eluent (95/5 to 93/7, *v*/*v*), to give *N1*-(2-hydroxyethyl) thymine as a white solid (300 mg, 1.76 mmol, 88%).

^1^H NMR (400 MHz, d6-DMSO) δ 11.18 (bs, 1H, H3), 7.43 (s, 1H, H6), 4.87 (bs, 1H, OH), 3.68 (t, J = 5.4 Hz, 2H, CH_2_-CH_2_-OH), 3.56 (q, J = 5.4 Hz, 2H, CH_2_-OH), 1.75 (s, 3H, CH_3-thymine_). ^13^C NMR (101 MHz, d6-DMSO) δ 164.9 (C4), 151.4 (C2), 142.9 (C6), 108.0 (C5), 59.1 (CH_2_-OH), 50.4 (CH_2_-CH_2_-OH), 12.4 (s, CH_3-thymine_). HRMS (ESI): *m*/*z* [M + H]^+^ calcd for C_7_H_11_N_2_O_3_: 171.0764 found 171.0764.

#### 2.3.4. Synthesis of N1-[(2-methacryloyloxy)ethyl] thymine (6)

To a suspension of *N1*-(2-hydroxyethyl) thymine (11) (200 mg, 1.18 mmol, 1 eq.) in anhydrous tetrahydrofuran (THF), triethylamine (0.66 mL, 4.72 mmol, 4 eq.) was added. The mixture was cooled at 0 °C and methacrylate chloride (0.14 mL, 1.42 mmol, 1.2 eq.) was added dropwise to the reaction. The mixture was stirred at 25 °C for 18 h. The volatiles were evaporated, and the expected *N1*-[(2-methacryloyloxy)ethyl thymine (6) was obtained as a withe powder (153 mg, 55%) by flash column chromatography on silica gel using PE/EtOac (1/1, *v*/*v*) as the eluent.

^1^H NMR (400 MHz, D6-DMSO) δ 11.25 (s, 1H, NH), 7.52 (s, 1H, H6), 6.01 (s, 1H, C=CH_2_), 5.68 (s, 1H, C=CH_2_), 4.29 (t, J = 5.1 Hz, 2H, CH_2_-O), 3.94 (t, J = 5.1 Hz, 2H, CH_2_-CH_2_-O), 1.84 (s, 3H, CH_3-acrylate_), 1.73 (s, 3H, CH_3-thymine_). ^13^C NMR (101 MHz, D6-DMSO) δ 166.2 (C=O_acrylate_), 164.2 (C4), 150.9 (C2), 141.7 (C6), 135.5 (C=), 126.1 (=CH_2_), 108.3 (C5), 61.8 (CH_2_-O), 46.2 (CH_2_-CH_2_-O), 17.9 (CH_3-acrylate_), 11.8 (CH_3-thymine_). HRMS (ESI): m/z [M + H]^+^ calcd for C_11_H_15_N_2_O_4_: 239.1026 found 239.1030.

### 2.4. Polymer Synthesis with Monomer (6) by Precipitation Polymerization

To an aqueous suspension of Tenofovir (1) (0.17 mmol, 1 eq.),2-(dimethylamino)ethyl methacrylate (4) was added and stirred until solubilization of the compounds (~2 min). Then, acetonitrile (MeCN) and the functional monomer *N1*-[(2-methacryloyloxy)ethyl thymine (6) (0.17 mmol, 1 eq.) were added. The resulting homogenous solution was stirred at room temperature for 4 h to form a pre-polymerization complex. Subsequently, 3.4 mmol (20 eq.) of EGDMA (cross-linker) and the initiator azobisisobutyronitrile (AIBN) (7.5 mg) were introduced into a round-bottom flask and degassed for 15 min with argon. After, the round-bottom flask was sealed and placed, overnight, in an oil bath at 70 °C without any stirring. After the polymerization took place, the polymeric precipitate was recovered by centrifugation at 20 °C, 12,000 RPM. Non-imprinted polymers (NIPs) were made in a similar manner, but with the omission of the Tenofovir template (1). The formulations are presented in Table 1.

### 2.5. Extraction of the Template (1)

Extraction of (1) was performed by consecutive ultrasound washings, using a mixture of methanol (MeOH) and acetic acid (AcOH) (9:1, *v*/*v*) for 1 h at 50 °C. After every washing step, the solution was replaced by a fresh mixture of solvents. The extracted concentration of Tenofovir was quantified by HPLC analysis to calculate the quantity of the antiviral remaining in the polymer. All Tenofovir solutions were analyzed on an Agilent 1260 Infinity LC System (Agilent Technologies, Les Ulis, France). The device is made up four modules, a high-pressure binary pump (G1311B), an autosampler (G1329B), a column oven (G1316A), and a UV–visible Diode Array Detector (G4212B) equipped with a Max-Light cartridge cell (1 μL volume, 10 mm cell path length). The separation was performed with the use of an Eclipse plus C18 analytical column (4.6 × 75 mm, 1.8 µm particle size) (Agilent Technologies, Les Ulis, France). The injection volume was 0.5 µL. The temperature of the column oven was set at 35 °C. The separation of Tenofovir is selective due to the fact that no other possible analytes, such as monomers, cross-linkers, and initiators used in the formulations, are co-eluted with antiviral. The mobile phase used in isocratic mode consisted of a mixture of 95% buffer (ammonium phosphate, pH 5) and 5% methanol, in *v*/*v* proportions, with a flow rate of 0.8 mL.min^−1^. Tenofovir showed a retention time of 2.5 min when monitored at its maximum absorbance wavelength equal to 260 nm (Figure S1). The calibration range used was linear (R^2^ = 0.9999) over the 0.004–2 mM concentration range (Figure S2).

### 2.6. Rebinding Studies of Tenofovir (1)

Batch adsorption experiments were used to estimate the binding capacity of the imprinted polymer. In an Eppendorf, 10 mg of MIP and NIP particles were thoroughly mixed with 1 mL of Tenofovir solution in a mixture of MeCN/H_2_O (1:1, *v*/*v*) at different concentrations (0.02–2 mM) and then thermostated at 25 °C for 16 h under continuous shaking (500 RPM). After the binding process was completed, the Eppendorf was used for centrifugation for 10 min at 10,000 RPM and the clear supernatant was removed. The free concentration of Tenofovir after rebinding was determined by the HPLC-UV method described in part 2.4. Three replicate extractions and measurements were performed for each concentration. The amount of Tenofovir bound per mass unit of polymers (Q_e_) was calculated according to Equation (1):

Q_e_ = (C − C_e_) × V/m (1)

where C is the concentration of Tenofovir before equilibrium, C_e_ is the free concentration of Tenofovir in the supernatant after equilibrium, V is the volume of the added solution, and m is the mass of the polymer.

Application of adsorption isotherm models allowed for the acquisition of important information concerning the strength of the interaction between the monomers and polymers, given by the association constant K_a_, the capacity of adsorption by “active” sites, given by Q_max_, and the m exponent of the Freundlich or Freundlich–Langmuir model.

The equations used to model the adsorption isotherms are as follows:

(2) $$\mathrm{Freundlich}:\;Q_{e}=K_{f}\times C_{e}^{m}$$

(3) $$\mathrm{Langmuir}:\;Q_{e}=\frac{Q_{max}\times K_{a}\times C_{e}}{1+K_{a}\times C_{e}}$$

(4) $$\mathrm{Freundlich}-\mathrm{Langmuir}:\;Q_{e}=\frac{Q_{max}\times {{(K}_{a}\times C_{e})}^{m}}{1+{{(K}_{a}\times C_{e})}^{m}}$$

Here, Q_e_: quantity adsorbed at equilibrium (mol/g); K_f_: Freundlich constant (L/g); m: Freundlich or Freundlich–Langmuir exponent; C_e_: solution concentration at equilibrium (mol/L); and Q_max_: maximum adsorption capacity (mol/g).

### 2.7. Kinetic Study of Tenofovir Release

The release studies of antivirals from imprinted and non-imprinted polymers were carried out placing 10 mg of loaded polymer particles in a dialysis bag (Spectra-Por^®^ Float-A-Lyzer^®^ G2, MW cut off 3.5–5 kDa, 1 mL, Dutscher, Bernolsheim, France) containing 1 mL of Phosphate-Buffered Saline (PBS) solution (pH 7.4). The dialysis bag was suspended in a flask containing 6 mL PBS buffer (pH 7.4), maintained at 25 °C or 37 °C. At required intervals, 50 µL of the buffer in the flask was collected for HPLC analysis. To maintain a constant release volume, the same volume of fresh PBS buffer solution, at the same temperature, was added immediately.

In order to investigate the kinetics of drug release in vitro and to understand the mechanism regulating its release from the materials, different kinetic models, such as zero-order, first-order, and Korsmeyer–Peppas, were used by applying the following Equations ((5)–(7), respectively) [35]:

(5) $$Q=Q_{0}+k_{0}\times t$$

(6) $$\mathrm{Ln}Q=\mathrm{Ln}Q_{i}+k_{1}\times t$$

(7) $$\frac{Q}{Q_{t}}=k\times t^{n}$$

where Q: quantity released of Tenofovir at time t; k: speed constant; Q_0_: initial amount released; Q_i_: initial amount in polymer; and Q_t_: total amount released.

## 3. Results

### 3.1. Solvent

**Figure 3:**
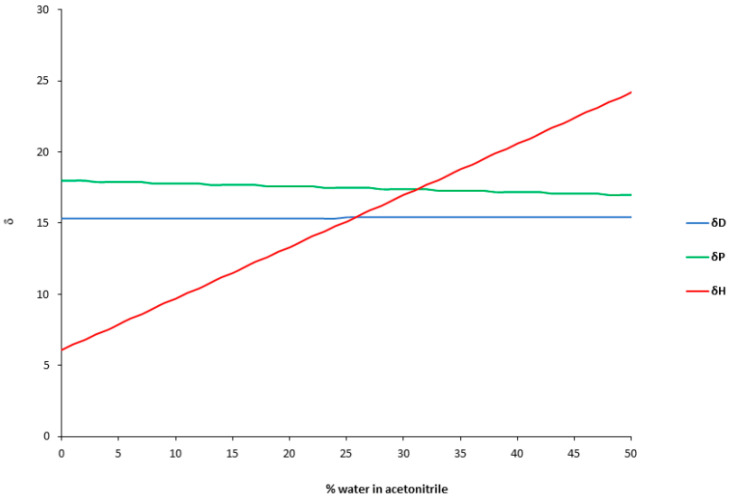
Evolution of Hansen’s parameters in function of percentage of water in acetonitrile (with calculations based on reference [38]).

In the development of imprinting polymers, the choice of solvent or mixture is crucial. In fact, the solvent must be able to solubilize all functionals monomers, the cross-linker, and the template. For biomedical application, the solvent should be biocompatible, show low toxicity, and be easily removable in the washing step. For polymerization precipitation, pure acetonitrile or acetonitrile in a mixture, most frequently with toluene, is the solvent of choice. Its use in non-covalent imprinting is interesting since it is a polar aprotic solvent which promotes hydrogen bonds between the template and functional monomers. Unfortunately, due to its physicochemical properties, especially its Log P of −1.12, calculated by MarvinSketch 23.17 (ChemAxon, Ltd., Budapest, Hungary), Tenofovir (1) is not soluble in this solvent. While water is a biocompatible solvent and can solubilize Tenofovir, this solvent cannot be used as a substitute for acetonitrile because its protic character destabilizes the hydrogen-bonded pre-polymerization complex [29]. Furthermore, the use of water in precipitation polymerization leads to the synthesis of irregular particles [36]. However, a binary mixture with a small amount of water can be used in imprinting polymerization and lead to regular particles. Kong et al. [37] demonstrated that an ethanol/water mixture produces spherical nanoparticles by precipitation polymerization at a water content of less than 12%. Solubility tests of Tenofovir on MeCN/H_2_O (1:1, *v*/*v*) showed that our template has a maximum solubility of 2 mM. Above a 50 vol.% of acetonitrile in the binary mixture, the solubility of Tenofovir becomes extremely low. Because of the maximum solubility in this binary mixture and the high percentage of water, a polar protic solvent which hinders the desired non-covalent intermolecular interactions, mainly hydrogens bonds, this mixture cannot be used. Among the three solubility parameters described by Hansen’s theory [38], which are δD (representing the non-polar interactions derived from the London dispersion force), δP (representing the polar interactions related to Keesom forces) and δH (related to the ability to form hydrogen bonds), it is this last parameter which rises sharply in the acetonitrile/water mixture as the percentage of water increases (Figure 3). This increase disfavors the base pairing interaction between adenine (Tenofovir) and thymine (monomer) nucleobases.

To overcome the poor solubility of Tenofovir in acetonitrile, an ionic pairing between the phosphonate moiety of Tenofovir and an appropriate monomer can increase its apolarity. Indeed, the phosphonate function of Tenofovir shows two pKa values (pKa_1_ = 1.31; pKa_2_ = 7.90) which are estimated by MarvinSketch 23.17 (Chemaxon, Ltd., Budapest, Hungary) and in accordance with those reported in the literature for alkylphosphonics acids [39]. In addition, in Figure S3 (SI) is plotted the percentage of different forms (neutral and protonated) of Tenofovir as a function of pH, showing that Tenofovir is monocharged for pH values between 3 and 7. As a consequence, functionals monomers containing amine moieties can be used to create ionic interactions.

### 3.2. Functionnal Monomers

Based on the chemical structure of Tenofovir, there are two potential sites for energetic interactions with ad hoc functional monomers, i.e., at the purine nucleobase adenine (acceptor and donor of hydrogen bond) and at the phosphonate moiety (ionic interactions).

#### 3.2.1. Choice of Monomer Ratio: Template for Ion Exchange Interaction

Concerning electrostatic interaction, required for solubilized Tenofovir in the MeCN/H_2_O mixture (9:1, *v*/*v*), the ratio of template/monomer is 1:1. According to Figure S3, Tenofovir is 97% monocharged at pH 6.4 and needs one equivalent of an amine monomer for ionic paring, provided Δpka > 4 [40]. Among the monomers with amine functionality, 2-(dimethylamino)ethylmethacrylate (7), showing a pKa of 8.42, estimated by MarvinSketch 23.17 (Chemaxon, Ltd.), has been used in imprinting polymers to recognize phosphophonate/phosphate moieties.

#### 3.2.2. Choice of Monomer Ratio: Template for Base Pairing

In order to recognize the nucleobase adenine, functionals monomers derived from uracil can be used. In fact, complementary base pairing by Watson–Crick interactions is well known and required for acyclic nucleoside phosphonates’ antiviral activities. Adenine has been shown to associate with thymine (or uracil) by creating two inter-molecular hydrogen bonding interactions. This kind of interaction has already been explored in polymeric fields, especially in imprinting polymers. This basic complementarity, via a thymine analog, the *N1*-(vinylbenzyl)thymine, was recently used in the design of two molecularly imprinted polymers by Mourão et al. [41], for adenosine monophosphate, and by Wei et al. [42], for 2′-deoxyadenosine. In both syntheses, the ratio of template/monomer was 1: 1, mimicking Watson–Crick interactions. Based on these results, we synthesized our monomer.

#### 3.2.3. Synthesis of Monomers (5) and (6)

**Scheme 1:**
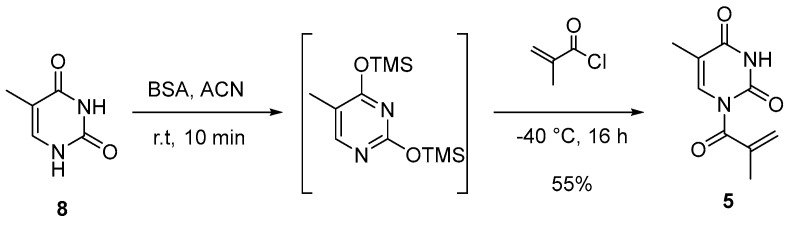
Synthesis of *N1*-methacryloyl thymine (5).

In the first instance, we decided to synthesize a thymine derivative by adding an acrylate function directly to the *N1* position. To increase the solubility of thymine and favor *N*-alkylation (versus *O*-alkylation), thymine was persilylated by reacting with *N*,*O*-bis(trimethylsilyl)acetamide (BSA). This silylated intermediate was then subjected to an *N*-acylation reaction in acetonitrile with methacryloyl chloride at −40 °C for 16 h. The *N1*-(methacryloyl)thymine (5) (Figures S4 and S5) was obtained in a 55% yield (Scheme 1). The low-temperature reaction favored the mono-substituted product over the di-substituted one, which was observed mainly at room temperature and reflux in dimethylformamide or acetonitrile.

**Scheme 2:**
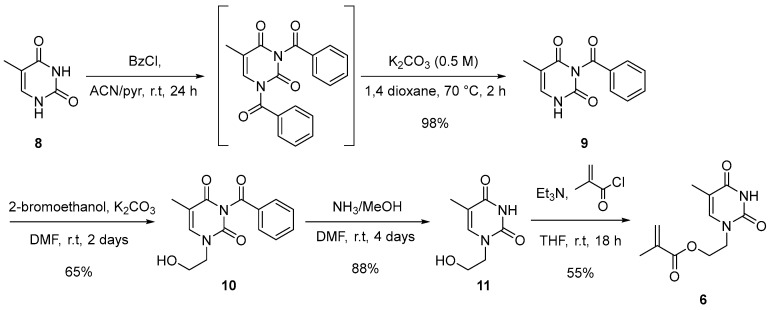
Synthesis of *N1*-[2-methacryloyloxy)ethyl] thymine (6).

However, this compound is highly unstable under water conditions (releasing the thymine) and it turned out that it could not be used as a functional monomer in our polymerization process performed in a mixture of MeCN/H_2_O (9:1, *v*/*v*). Thus, to overcome this problem, we decided to synthesize *N1*-[(2-methacryloyloxy)ethyl thymine (6) (Figures S6 and S7), which possess a non-hydrolysable ethyl linker between the pyrimidine base and the polymerizable function. This linker can also provide flexibility during the creation of the cavities in imprinting polymerization. The synthesis of 6 is outlined in Scheme 2. After the dibenzoylation of thymine at *N1* and *N3*, treatment with a solution of potassium carbonate in 1,4-dioxane at 70 °C for 2 h resulted in selective *N1* deprotection and gave the expected *N3*-benzoyl thymine (9) (Figures S8 and S9) in a 98% yield.

Following the procedure of Kitamura et al. [43], the *N3*-benzoylthymine (8) was subjected to *N*-alkylation using 2-bromoethanol and potassium bicarbonate in dimethylformamide at room temperature for 2 days to give compound (10) (Figures S10 and S11) in a 65% yield. Removal of the benzoyl group with ammonium methanolate afforded the *N1*-(2-hydroxyethyl)-thymine (11) (Figures S12 and S13) in an 88% yield. The reaction of *N*-acylation between compound (11) and methacryloyl chloride in the presence of triethylamine in tetrahydrofuran at room temperature for 18 h gave the expected *N1*-(2-methacryloyloxy)ethyl] thymine (6) in a 55% yield.

### 3.3. Imprinted Polymer Synthesis

**Figure 4:**
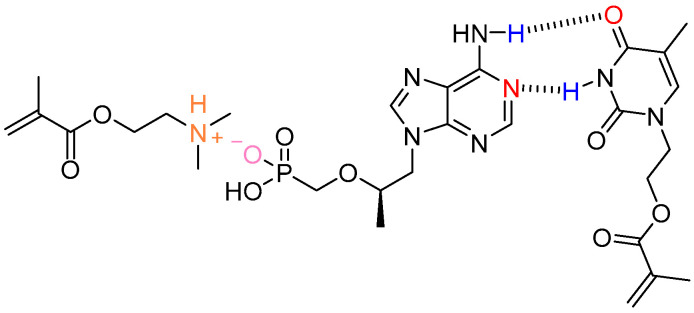
Putative pre-polymerization complex between Tenofovir (1), DMAEMA (4), and *N1*-[(2-methacryloyloxy)ethyl] thymine (6).

As previously mentioned, an ionic pairing interaction is required to solubilize Tenofovir in a mixture of MeCN/H_2_O (9:1, *v*/*v*). A pre-polymerization complex (Figure 4) is formed by solubilized Tenofovir in water with 2-(dimethylamino)ethyl methacrylate (DMAEMA) (4), followed by the addition of acetonitrile and *N1*-[(2-methacryloyloxy)ethyl] thymine (6). The cross-linker chosen is ethylene glycol dimethacrylate (EGDMA) (7), which belongs to the same acrylate family. It is a biocompatible cross-linker, one of the most widely used in MIP design and soluble in our MeCN/H_2_O mixture. This cross-linker was also chosen to minimize hydrophilic interactions with the template. In addition, the use of high cross-linking degrees can lead to better recognition performance and a slower release kinetic in molecularly imprinted polymers compared to non-imprinted materials.

The formulation of the MIP is described in Table 1. A non-imprinting polymer was synthesized in parallel under the same conditions as the MIP, but in absence of Tenofovir, to serve as a control (blank). Theoretically, due to the absence of the template, the NIP had only nonspecific sites, different to the MIP which represented nonspecific sites and specific recognition sites. These sites are able to recognize the shape, size, and chemical functionalities of the target molecule. Therefore, the NIP was necessary to characterize the selectivity of the MIP for the template, Tenofovir.

### 3.4. Optimization of the Template Extraction

After polymerization, a washing step of the polymeric materials was conducted to eliminate the residues of reagents (monomers, cross-linker, etc.) and extract the template from the MIP cavities. Among the several washing methods cited in the literature [44], the ultrasound method was chosen. It has already proved its worth in the extraction of nucleosides, like ribavirin or adenosine 5′-monophosphate. To ensure the conformity of our analyses, the MIP and NIP washes were carried out under the same conditions. In order to break the ionic interaction and hydrogen bonds, our washing solution was composed of an MeOH/AcOH mixture (9:1, *v*/*v*), one of the most widely used mixtures for extracting small organic molecules acting as templates [45]. Every hour, the washing suspension was centrifugated and the washing liquid was changed. A few microliters of the supernatant was injected by HPLC to determine the extracted amount of Tenofovir every hour. After a number of washes, no more Tenofovir was extracted and three washes with the MeOH/H_2_O solution (9:1 *v*/*v*) were performed to remove acetic acid from the medium.

### 3.5. Physical Characterisation

**Figure 5:**
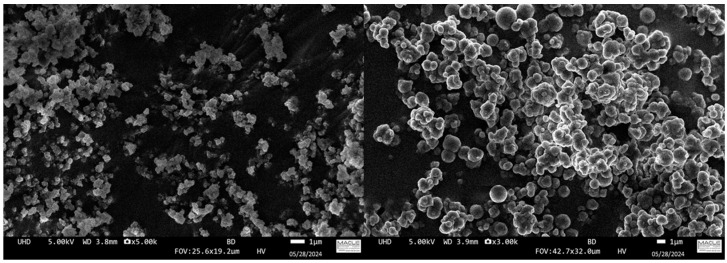
Scanning electron microscopy analysis of MIP (**left**) and NIP (**right**).

Scanning electron microscopy was used to determine the morphology of the synthesized polymers, both the MIP and NIP (Figure 5). These analyses were performed at Platform MACLE-CVL, using the apparatus JEOL IT800SHL FEGSEM (Field Emission Scanning Electron Microscope (Schottky)).

**Figure 6:**
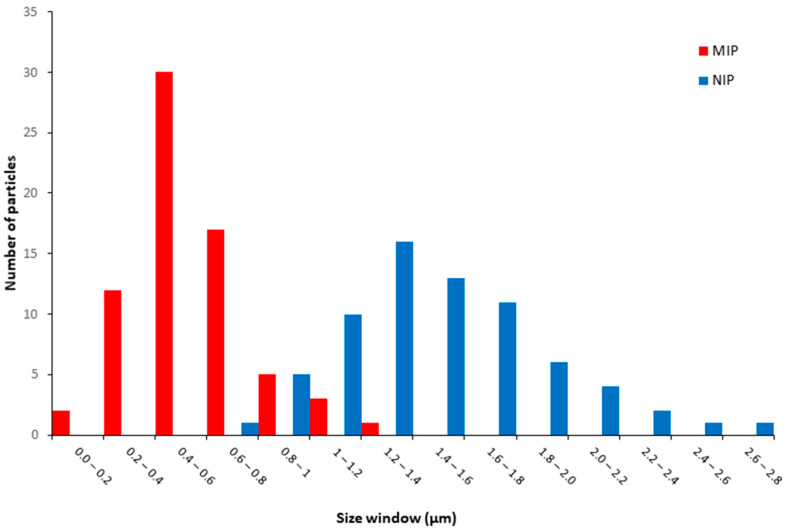
Histogram of particle size distribution for MIP material (red bars) and NIP material (blue bars).

Figure 5 illustrates the morphological analysis of the two polymers using scanning electron microscopy (SEM). The micrographs obtained revealed no significant differences in textural properties; however, there was a change in the mean particle size, as shown in Figure 6. The mean size of MIP particles was 0.618 ± 0.187 µm, which was lower than that of NIP ones, calculated as 1.261 ± 0.238 µm. All size measurements were carried out on 75 particles. The range of size values was lower for the MIP comparatively to the NIP, with 1.179 µm and 2.042 µm for the MIP and NIP material, respectively. The use of a porogen, in the form of a mixture of water and predominantly acetonitrile, appears to be effective in the synthesis of polymeric particles imprinted by precipitation with a hydrophilic template that is not soluble in the organic solvent alone. But the template could play a role in the morphology of MIP particles, as shown here by the mean size being lower for the MIP than the NIP (Figure 6).

### 3.6. Binding Isotherms of Tenofovir

**Figure 7:**
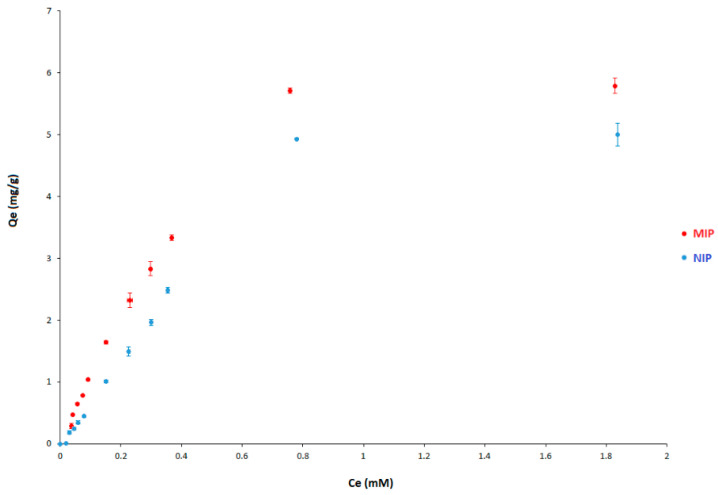
Adsorption isotherms of Tenofovir for MIP and NIP (error bars represent +/− Standard Deviation calculated for 3 values of Ce and Qe for each point of the curves).

Adsorption studies of Tenofovir on MIP submicroparticles and NIP microparticles were performed by batch. It is well known that the recognition and selectivity of a MIP for its template is enhanced when the recapture solvent is identical to the polymerization solvent. However, due to the low solubility of Tenofovir in a mixture containing a large proportion of acetonitrile, we chose to perform these adsorption studies in a mixture of MeCN/H_2_O (1:1, *v*/*v*). Adsorption isotherms were obtained by plotting the adsorbed quantity (Qe) versus the equilibrium concentration (Ce) of Tenofovir. The polymer (10 mg) was brought into contact with 1 mL of Tenofovir solution of different concentrations ranging from 0.004 to 2 mM (the concentration of 2 mM corresponds to the maximum solubility of antiviral in this medium). The contact period lasted 16 h to ensure maximum adsorption at equilibrium. Adsorption isotherms as a function of equilibrium concentration Tenofovir for MIP and NIP are shown in Figure 7.

The maximum adsorption capacity Q_max_, the association thermodynamic constant K_a_, and the exponent m are summarized in Table 2. The presence of specific cavities in the MIP is usually checked by dividing the amount of template bound on the MIP by that bound on the NIP at a given concentration. The higher the ratio, named the imprinting factor (IF), reaches above 1, the more evidence there is of molecularly imprinted cavities within the MIP. Because, at the value of 1, the MIP has the same behavior of adsorption to the reference material, the non-imprinted polymer, the presence of a population of specific recognition sites, due to the template in the synthesis medium, is not demonstrated.

According to results of Table 2, the best fit is obtained with the theoretical Freundlich–Langmuir model for the MIP and NIP. In order to determine the correct correlation between the theoretical and experimental models, several statistical tests were considered, such as the coefficient of determination R^2^, which should be as close to 1 as possible, the Fisher F_value_ test, which should be as high as possible, the Chi-square test (χ^2^), which should give a number as small as possible, and the Residual Sum of Squares (RSS), which should show the lowest possible calculation result. These data are shown in the Table 2.

Adsorption isotherms are used to understand the equilibrium mechanisms between adsorbate, Tenofovir, and adsorbent, MIP or NIP, by plotting the quantity of molecule adsorbed as a function of its equilibrium concentration. Generally, three models are used to simulate these isotherms in studies of molecularly imprinted materials. There are the empirical Freundlich model, the theoretical Langmuir model, and a combination of the two equations, resulting in the empirical Freundlich–Langmuir model. The first model requires two assumptions: the molecules are distributed on the surface according to Boltzmann’s law and the adsorption energies are much higher than the thermal energy. This model makes it possible to explain the adsorption equilibrium for adsorbent materials presenting a heterogeneous surface in terms of heat of adsorption. The assumptions of Langmuir’s model are that adsorption must be monolayer, that all sites are equivalent in terms of interaction energy, and that there are no lateral interactions between the adsorbed molecules. The Freundlich–Langmuir equation reduces to the Freundlich equation at low solute concentrations and, like the Langmuir equation, exhibits a finite saturation limit at sufficiently high concentrations. Figure 7 shows that the curves representing adsorbed quantities per unit mass of material (Q_e_) for MIPs and NIPs are not merged, and that the MIP curve shows a higher Q_e_ than the NIP one over the entire Tenofovir concentration range. This phenomenon indicates an imprinting effect. Both isotherms have a peculiar shape, corresponding to the “S” (Sigmoidal) type with an inflection point. The sigmoid shape of an adsorption isotherm is generally regarded as indicative of a cooperative adsorption effect [46]. This is confirmed by the fact that the exponential factor associated with equilibrium solution concentrations in the Freundlich–Langmuir model is higher than 1 (Table 2). When this number is equal to 1, the model is equivalent to the theorical Langmuir model, and if the exponent n associated with the concentration is less than 1, it is instead linked to a Gaussian distribution of sorption/binding sites by energy. Consequently, the exponent m (>1) can be interpreted as the degree of cooperativity of the adsorption process [47]. In this way, n takes on a physical meaning, as the number of molecules cooperating during absorption. In fact, the sorption of the first molecule of Tenofovir promotes the subsequent adsorption of other molecules. In some cases, the exponent can represent the formal number of molecules cooperating in such a ‘collaborative’ interaction. [48]. This phenomenon has been reported several times in literature in the case of the adsorption of nucleotides, of which Tenofovir is an analogue [49,50,51].

**Figure 8:**
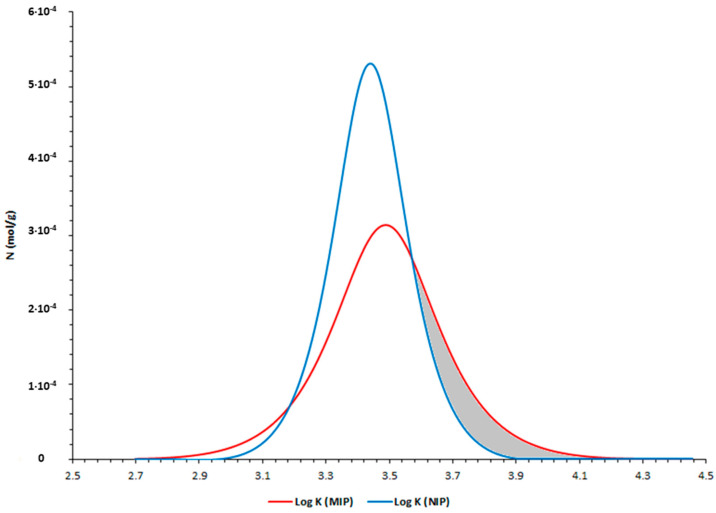
Affinity distributions of the MIP and NIP, red and blue curves, respectively, based on the Freundlich–Langmuir model (Equation (4)).

Here, the exponent found for the NIP is almost equal to 2. If we assume that this number reflects the number of molecules interacting together on the polymer surface, we could assume that Tenofovir is anchored to the surface in pairs. The first molecule bonds via the phosphonate function to the ammonium moiety associated with the DMAEMA monomer forming the material. This is because the energy of the ionic interaction is higher than a hydrogen bond, which is relatively unfavorable in a semi-aqueous medium. A second molecule then joins the first by associating with the nucleic bases (adenosine) of Tenofovir on the basis of base-stacking, as was discussed in a previous work on the adsorption of nucleotides on minerals [49]. The capacity seems to be related to non-specific interactions (no specific cavities), directly linked to the accessible ion exchange functions. Of course, the thymine-derived monomer plays a role in adsorption, but not to the same extent as on the MIP. The Gaussian curve for the affinity distribution, N(K) versus LogK (Figure 8), of the NIP is tighter than that of the MIP, with an apex placed at a value of LogK lower than that of the MIP. This indicates less heterogeneity in terms of adsorption energy in the NIP, but with an average value less pronounced than that of the MIP. For the MIP, the affinity distribution curve, expressed by a Gaussian distribution, is wider than that for the NIP. The decreasing part, at high levels of LogK, corresponds to the part of the isotherm corresponding to low concentrations, where the high-affinity binding sites are preferentially occupied [52]. This demonstrates the presence of specific cavities within the imprinted material (grey area shown in Figure 7 between the MIP and NIP affinity distribution curves, in red and blue colors, respectively).

Finally, the imprinted material showed a greater capacity both in terms of moles of Tenofovir adsorbed per unit mass of material (22.7 µmol/g) than the non-imprinted one (18.9 µmol/g), and in terms of the adsorption constant, 3079 versus 2754.

Particles synthesized in the presence of the template fully demonstrated the presence of specific recognition cavities for Tenofovir, as the imprinting factors (IFs) are 5.48, 1.61 and 1.23 for concentrations of this antiviral equal to 1.95 · 10^−5^, 1.83 · 10^−4^ and 2 · 10^−3^ mol·L^−1^, respectively.

These results are confirmed by the release study where the total loadings of the materials, MIP and NIP, are initially found to be 2.75 · 10^−5^ mol·g^−1^ and 2.18 · 10^−5^ mol·g^−1^, respectively, and correlate with theoretical ones (calculated by Freundlich–Langmuir model, Equation (4)). Moreover, due to the difference in association constants favorable to the MIP, and so to the IF, Tenofovir should show a slower release profile than the NIP.

### 3.7. Kinetics of the Tenofovir Release

**Figure 9:**
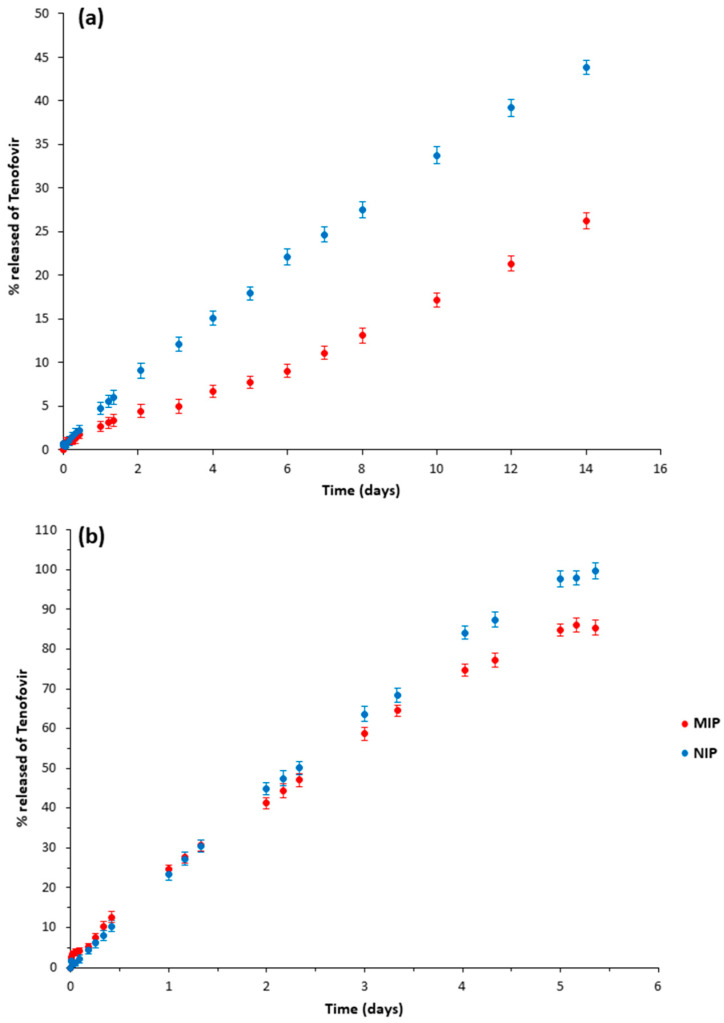
Release of Tenofovir (expressed as % of initial mass adsorbed in each material) as a function of time in Phosphate-Buffered Saline solution at 25 °C (**a**) and 37 °C (**b**).

A kinetic study of Tenofovir release was performed on the synthesized MIP and NIP. Thus, 10 mg of each synthetized material was loaded by using 1 mL of a solution of Tenofovir at 2 mM in an MeCN/H_2_O mixture (1:1, *v*/*v*). This release study was conducted in phosphate buffer saline (pH 7.4, 0.01 M) at 25 °C and 37 °C. HPLC analysis of the release medium at different times yielded the quantities of Tenofovir reloaded as function of time. The ratio of the released mass of Tenofovir to the initial mass of Tenofovir contained in the polymers as function of time is plotted in Figure 9 at 25 °C and 37 °C, respectively.

At 25 °C, the obtained curves show a sustained release of Tenofovir which is more pronounced in the imprinting polymer. Indeed, after 2 weeks, the cumulative percentage of Tenofovir release is 43.85 ± 1.77% and 26.19 ± 1.67% for the NIP and MIP, respectively. The NIP seems to correlate to an affine line. We applied two models, the kinetic models of zero-order and first-order. This first model describes a release rate of the active ingredient that is independent of its concentration. By applying the zero-order and first-order models, coefficients of determination (R^2^) were obtained using linear regression (0.9953 for zero-order and 0.9995 for first-order kinetics). This coefficient is greater for the first-order model. Thus, Tenofovir release from the non-imprinted material follows the first-order model, with a constant rate of 0.0017 h^−1^.

The MIP profile appears to be triphasic and does not correlate with any classical model. The profile shows a slight flattening after 2 days. This phenomenon may be related to the hydration of the polymer. At the beginning, the release of Tenofovir results from non-specific interactions on the particle surface. Then, as the imprinting polymer is hydrophobic due to the high cross-linking density of EDGMA, the diffusion of water takes time to reach the Tenofovir retained more deeply in the polymer matrix. After a few days, when the polymer is fully hydrated, the release of Tenofovir increases rapidly. This phenomenon may be due to swelling or degradation of the polymer matrix [53].

At 37 °C, the obtained curves showed a sustained release of Tenofovir for both imprinting and non-imprinting faster than at 25 °C. In fact, a total delivery (99.71 ± 1.92%) is observed in 6 days for the NIP (only 22.79 ± 1.67% at 25 °C) while the MIP released 85.42 ± 1.84% in the same time (only 9.03 ± 0.84% at 25 °C). These results showed that temperature influenced the kinetic release of Tenofovir. Indeed, higher temperatures accelerate the diffusion process, resulting in the polymer being quickly and thoroughly hydrated. Thus, Tenofovir retained in the cavities will be released more rapidly. The interaction between hydrophobic chains inside the polymeric matrix, due to dispersion forces, is reduced at 37 °C, facilitating solvent entry and disturbing the cavity’s integrity and pushing out the Tenofovir complexed in these “molecular imprints”. But, also, by increasing the temperature of medium, the solubility of Tenofovir in the solvent is increased, and the forces of attraction, ionic exchange, hydrogen bonds, and probably pi-stacking, existing between the matrix and adsorbed Tenofovir, are significantly reduced.

To describe the release phenomenon, we applied the Korsmeyer–Peppas [54] (Equation (7)) model, which already been used to describe the release of antiviral drugs [55,56]. The results of the nonlinear regression are summarized in Table 3.

In this model, the equation contains a parameter identified by the letter n, which is important in the understanding of the release mechanism(s) of the active ingredient from a DDS. Indeed, if n has a value less than 0.5, then the diffusion mechanism of drug in the DDS follows the Fick model and the diffusion amplitude is the same in all directions. The solvent penetrates very easily in the matrix, dissolving the drug and releasing it. The accepted predominant release mechanism is diffusion. If the n value is higher than 1, then the mechanism of release is named “Super Case II transport”. The drug transport mechanism is mainly dominated by the relaxation process of DDS polymer chains, and not diffusion, due to swelling, dissolution, or erosion mechanisms. This phenomenon is obtained generally with a hydrogel DDS in water, because the polymeric chains are hydrophilic. But, if the n value ranges from 0.5 to 1, then the release mechanism is named “Non-Fikian” [57]. This was the case for both materials, the imprinted and non-imprinted one. The release mechanism is therefore governed simultaneously by Tenofovir and aqueous solution diffusion within the polymer matrix and by polymer “relaxation”. This term conceals several phenomena such as swelling and degradation of the DDS polymeric matrix. The n values obtained for both the MIP and NIP were very close, indicating that the diffusion and “relaxation” rates were comparable. These results were consistent with similar kinetic profiles shown in Figure 9b, when the temperature of incubation was 37 °C. But despite the fact that the release profiles of the two materials are almost identical at body temperature, the MIP released 15% less than the NIP after 6 days, while the NIP completely released the adsorbed amount of Tenofovir in the same time. At this elevated temperature, the rate of release of the imprinted polymer particles is nevertheless significantly low and allowed us to assert that this DDS results in a sustained release profile. This fact was even more marked at 25 °C. Both profiles were very dissimilar at this temperature. Even after 2 weeks of release, the imprinted polymer desorbed only 26% of its loaded Tenofovir. The release rate of the MIP was therefore much lower than that of the NIP, demonstrating the impact of molecular imprinting on the DDS release profile. The energy of “complexation” of Tenofovir inside the cavities was greater than that involved in adsorption of the same molecule on the surface of the non-imprinted material. And the Tenofovir was held in its cavity by the specific interactions existing between the polymer and the compound chemical functions. But the molecule was also retained by shape and size recognitions, which slowed or blocked its possible exit even when its chemical function interactions were reduced. So, at 25 °C, the movement of the polymeric chains that formed these cavities was lower than at 37 °C, and the three-dimensional destructuring of these molecular pockets was less marked. We could not investigate further which type of phenomenon was most present; maybe slight swelling might be more likely than the hydrolysis of the acrylate matrix under these pH and temperature conditions. As a result, antiviral release from the MIP was much slower than from the NIP. Thus, if this type of DDS requires preparation, such as dispersion in a buffered phase prior to administration, then it is demonstrated that the percentage of therapeutic agent lost in the solution during this step will be very low. Once charged, the imprinted polymer selective to Tenofovir could be stored for a long time in a dry form or in solution before use at room temperature or lower.

## 4. Conclusions

Using a non-covalent approach, we synthesized via polymerization by precipitation a MIP of Tenofovir (1) using an MeCN/H_2_O (9:1, *v*/*v*) mixture. From a large variety of potential functional monomers, we chose to favor (i) an ionic interaction between a negatively charged phosphonic acid moiety and DMAEMA and (ii) a Watson–Crick-type interaction with the nucleobase of in-house-synthesized monomers *N1*-methacryloyl thymine (5) and *N1*-[(2-methacryloyloxy)ethyl] thymine (6). Unfortunately, due to the low stability of (5) in water, only monomer (6) was used. The adsorbent properties of the materials were assessed by determining the Tenofovir adsorption isotherms, and the Freundlich–Langmuir model indicates the best correlation for the MIP and NIP. But the imprinted material possessed a total number of binding sites of 2.27 · 10^−5^ mol·g^−1^, while the capacity of the NIP is lower, 1.89 · 10^−5^ mol/g. The presence of specific cavities is demonstrated for the MIP, with an IF equal to 5.5 at 1.95 · 10^−5^ M, with a Ka higher for the MIP than NIP, 3079 versus 2754, respectively. We studied the release of Tenofovir from our synthetic material at two different temperatures. Although there is no significant difference in Tenofovir release between MIP and NIP at 37 °C, our MIP demonstrates a slow and controlled release of Tenofovir, with 41.25 ± 1.38% released in just over 2 days. Furthermore, since the release kinetics of the MIP at 25 °C are extremely slow, 26.17 ± 1.67% in 2 weeks, our filled materials can be prepared and stored at room temperature or at 4 °C for several days or weeks before administration without losing their potency.

## Figures and Tables

**Table 1:** Formulation used in synthesis of molecularly imprinted polymers (MIPs) selective to Tenofovir and non-imprinted polymers (NIPs).

	Tenofovir	DMAEMA ^a^	Molecule 6	Ratio (T:M1:M2) ^b^	EGDMA ^c^	AIBN ^d^	MeCN ^e^	H_2_O
MIP	50 mg	29 µL	42 mg	1:1:1	658 µL	7.5 mg	68 mL	8 mL
NIP	/	29 µL	42 mg	0:1:1	658 µL	7.5 mg	68 mL	8 mL

^a^ DMAEMA is 2-(dimethylamino)ethyl methacrylate; ^b^ T is Tenofovir (Template), M1 is monomer 1, DMAEMA and M2 are monomer 2, the compound (6), or *N1*-[(2-methacryloyloxy)ethyl thymine; ^c^ EGDMA is ethylene glycol dimethacrylate; ^d^ AIBN is azobisisobutyronitrile; and ^e^ MeCN is acetonitrile.

**Table 2:** Values of affinity constants (Ka; L·mol^−1^), number of active sites (N; mol·g^−1^), coefficient of determination (R^2^), Fisher statistical test value (F_value_), Chi-square value (χ²), and Residual Sum of Squares (RSS) (obtained by applying the different adsorption isotherm models to the MIP and NIP materials).

		Q_max_	Ka	m	R²	χ²	F_value_	RSS
Freundlich	MIP	1.51 · 10^−5^	3758.34	0.502	0.87033	5.84 · 10^−12^	291.58	1.81 · 10^−10^
	NIP	ND *	ND	ND	ND	ND	ND	ND
Langmuir	MIP	2.74 · 10^−5^	1973.30	/	0.963	1.71 · 10^−12^	1003.92	5.46 · 10^−11^
	NIP	2.59 · 10^−5^	1373.01	/	0.946	2.04 · 10^−12^	545.31	6.53 · 10^−11^
F-L	MIP	2.27 · 10^−5^	3079.22	1.452	0.980	9.04 · 10^−13^	1272.70	2.80 · 10^−11^
	NIP	1.89 · 10^−5^	2754·26	1.998	0.982	6.77 · 10^−13^	1117.30	2.10 · 10^−11^

* Not determined (Fit Status is Failed).

**Table 3:** Values of coefficient of determination (R^2^), speed constant (k; h^−n^), and n (release exponent) obtained by applying Korsmeyer–Peppas model to the MIP and NIP materials.

	Korsmeyer-Peppas Model		
Materials	R^2^	k (h^−n^)	n
MIP	0.9990	1.91	0.80
NIP	0.9994	1.24	0.92

## Data Availability

The data presented in this study are available on request from the corresponding author.

## Supplementary Materials

The following supporting information can be downloaded at: https://www.mdpi.com/article/10.3390/pharmaceutics16070965/s1, Figure S1: Chromatogram of Tenofovir; Figure S2: HPLC-UV calibration range for Tenofovir; Figure S3: Percentage of predominantly forms of Tenofovir in function of pH; Figure S4: ^1^H NMR spectra of compound (5); Figure S5: ^13^C NMR spectra of compound (5); Figure S6: ^1^H NMR spectra of compound (9); Figure S7: ^13^C NMR spectra of compound (9); Figure S8: ^1^H NMR spectra of compound (10); Figure S9: ^13^C NMR spectra of compound (10); Figure S10: ^1^H NMR spectra of compound (11); Figure S11: ^13^C NMR spectra of compound (11); Figure S12: ^1^H NMR spectra of compound (6); Figure S13: ^13^C NMR spectra of compound (6).
