# Supplementary material for: Molecularly Imprinted Polymeric Nanoparticles as Drug Delivery System for Tenofovir, an Acyclic Nucleoside Phosphonate Antiviral
Source: Pharmaceutics. 2024 Jul 21;16(7):965. doi: 10.3390/pharmaceutics16070965 (PMC11280436; doi:10.3390/pharmaceutics16070965)
Supplement: Supplementary file 1 [file pharmaceutics-16-00965-s001.zip › pharmaceutics-3065925-supplementary.pdf]

Supplementary Materials:

Molecularly Imprinted Polymeric Nanoparticles as Drug Delivery System for Tenofovir, an Acyclic Nucleoside Phosphonate Antiviral

Chromatogram of Tenofovir (**Figure S1**)

HPLC-UV calibration range for Tenofovir (**Figure S2**)

Percentage of predominantly forms of Tenofovir in function of pH (**Figure S3**)

^{1}H and ^{13}C NMR spectra of compound 5 (**Figures S4 and S5**)

^{1}H and ^{13}C NMR spectra of compound 6 (**Figures S6 and S7**)

^{1}H and ^{13}C NMR spectra of compound 9 (**Figures S8 and S9**)

^{1}H and ^{13}C NMR spectra of compound 10 (**Figures S10 and S11**)

^{1}H and ^{13}C NMR spectra of compound 11 (**FigureS S12 and S13**)

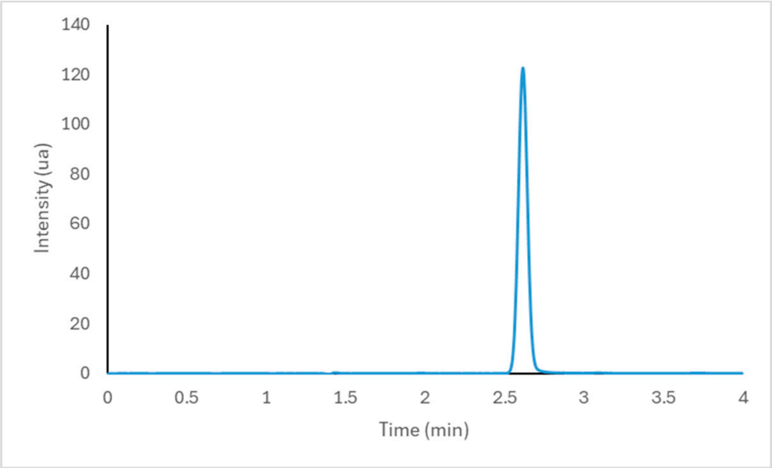

Figure S1: Chromatogram of Tenofovir (Column Eclipse plus C18, 4.6 x 75 mm, 1.8 µm, T= 35 °C, mobile phase: ammonium phosphate, pH 5/MeOH (95/5, v/v), Flow rate = 0.8 mL.min^{-1}, λ = 260 nm

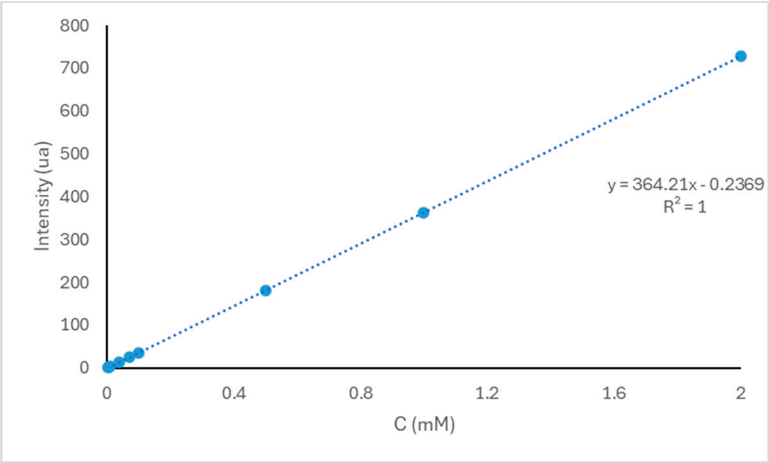

Figure S2: HPLC-UV calibration range (0.004 to 2 mM) for Tenofovir

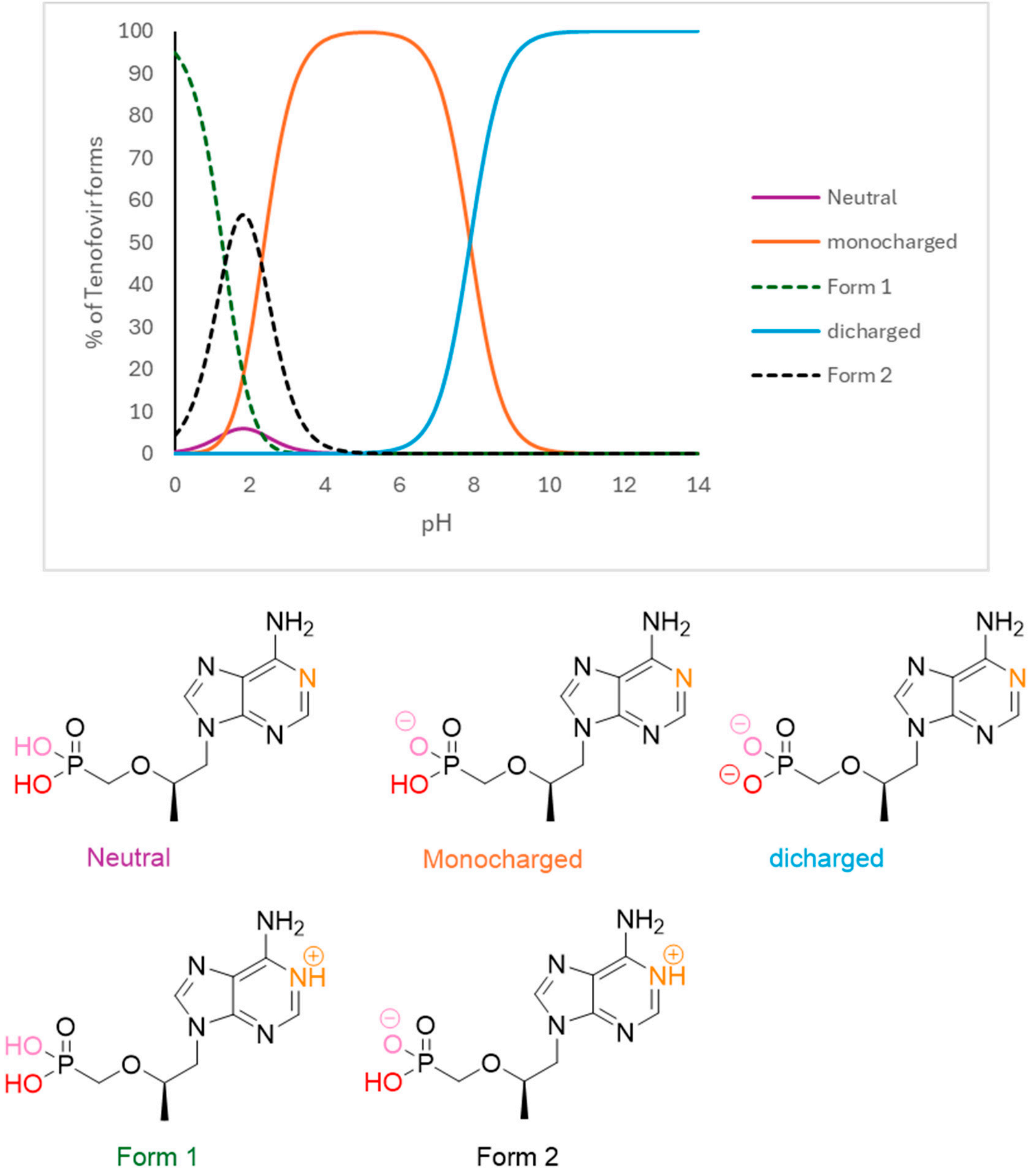

Figure S3: Percentage of predominantly forms of Tenofovir in function of pH

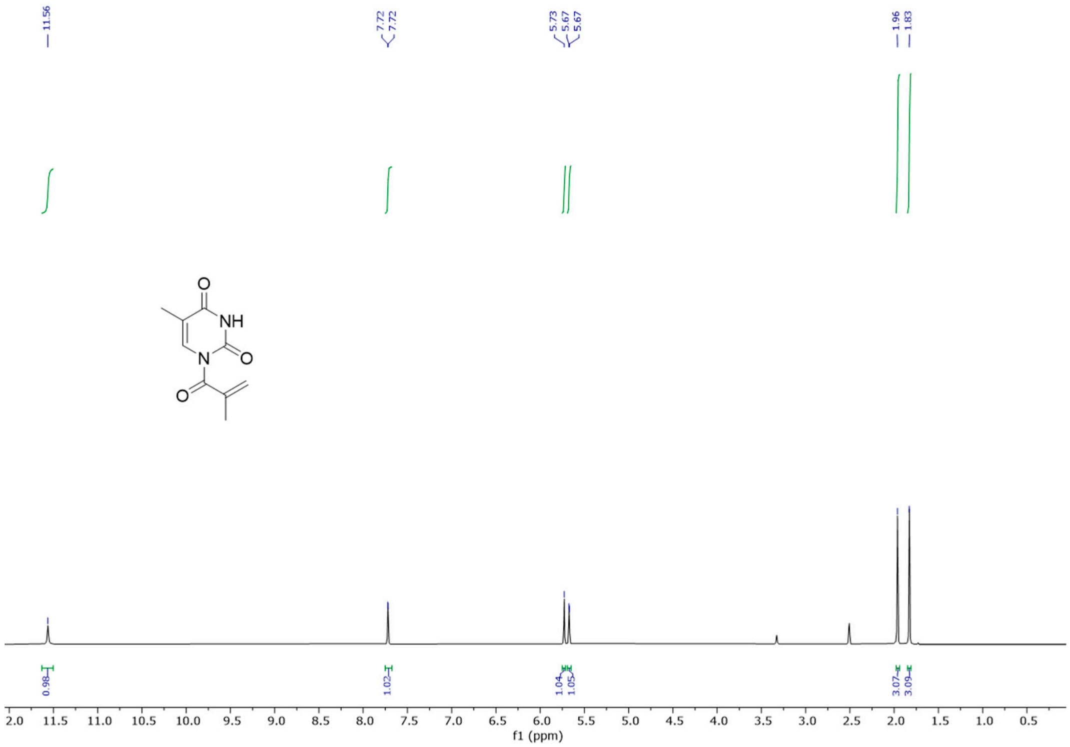

Figure S4: ^{1}H NMR of compound 5 (d6-DMSO)

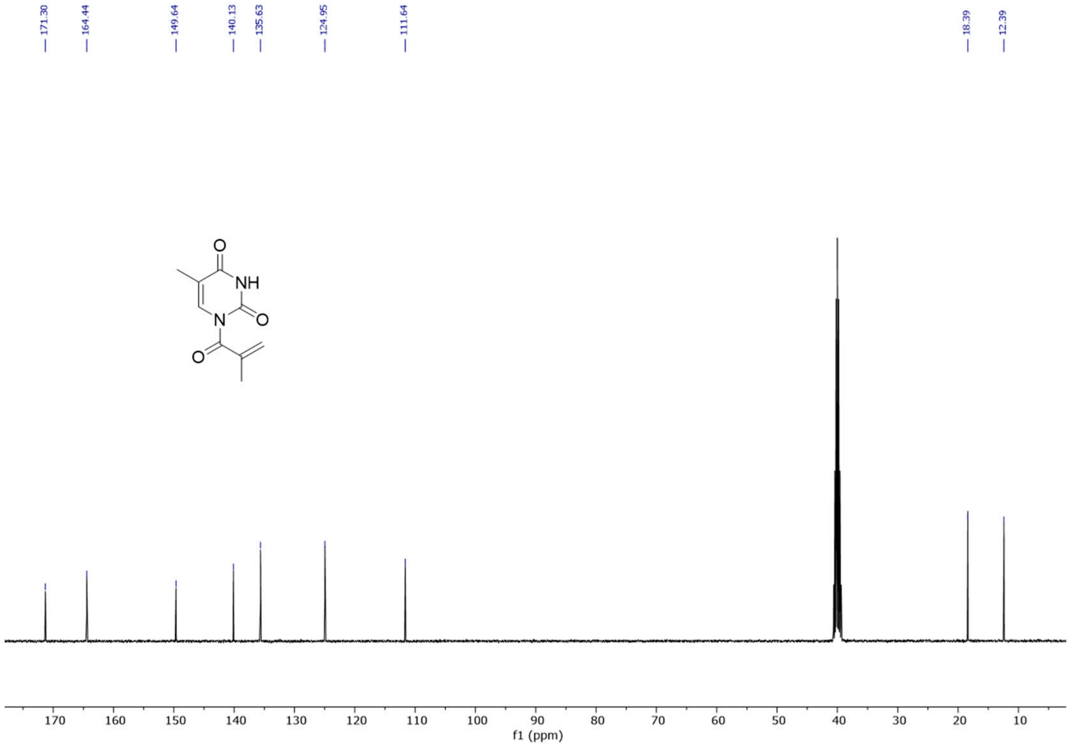

Figure S5: ^{13}C NMR of compound 5 (d6-DMSO)

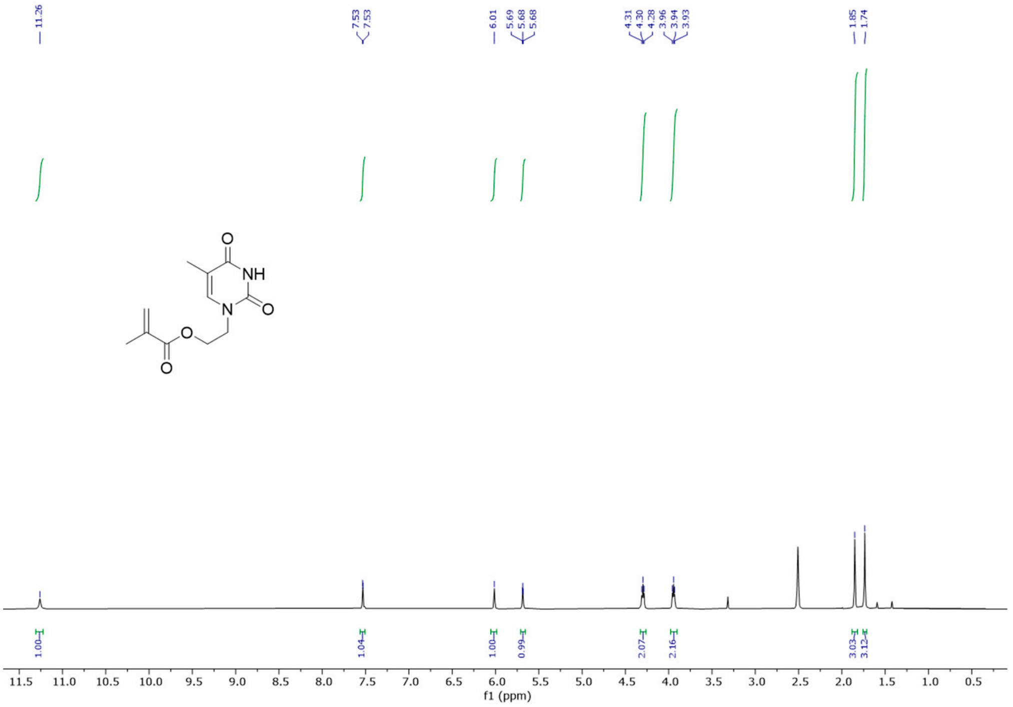

Figure S6: ^{1}H NMR of compound 6 (d6-DMSO)

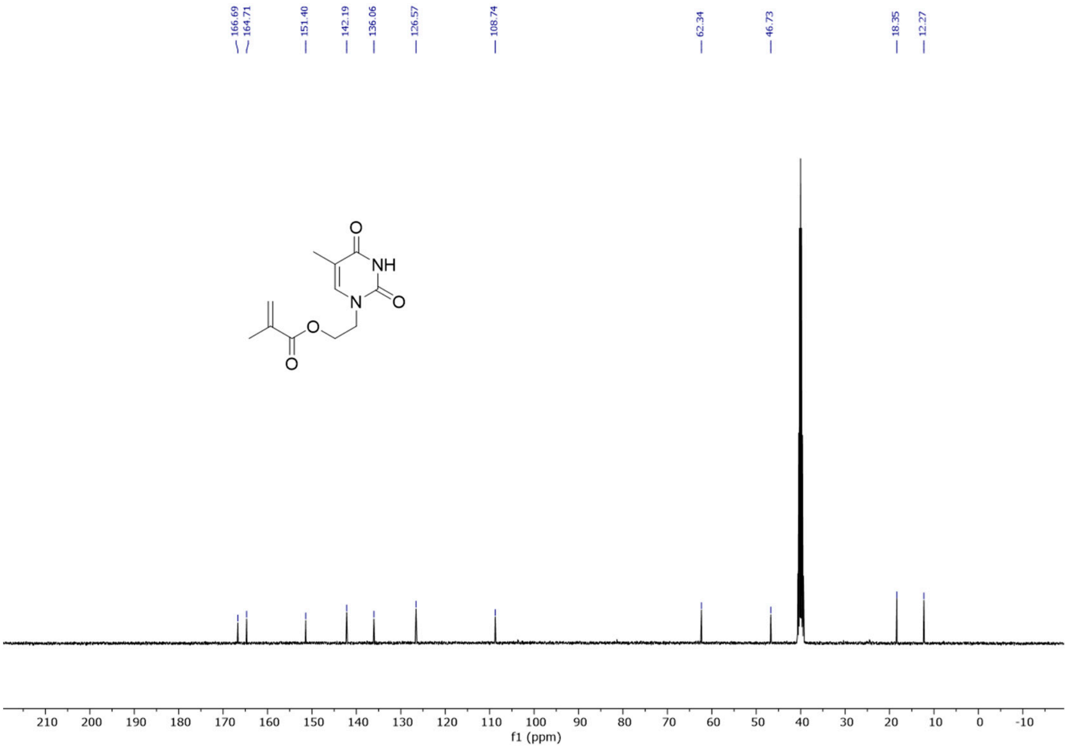

Figure S7: ^{13}C NMR of compound 6 (d6-DMSO)

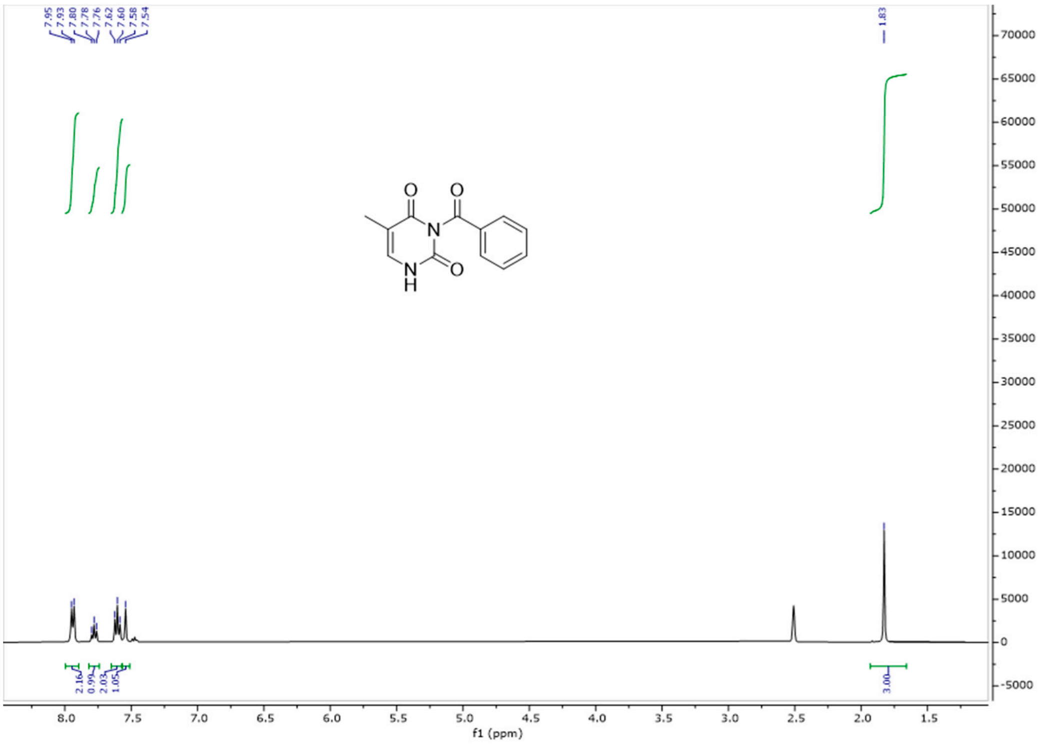

Figure S8: ^{1}H NMR of compound 9 (d6-DMSO)

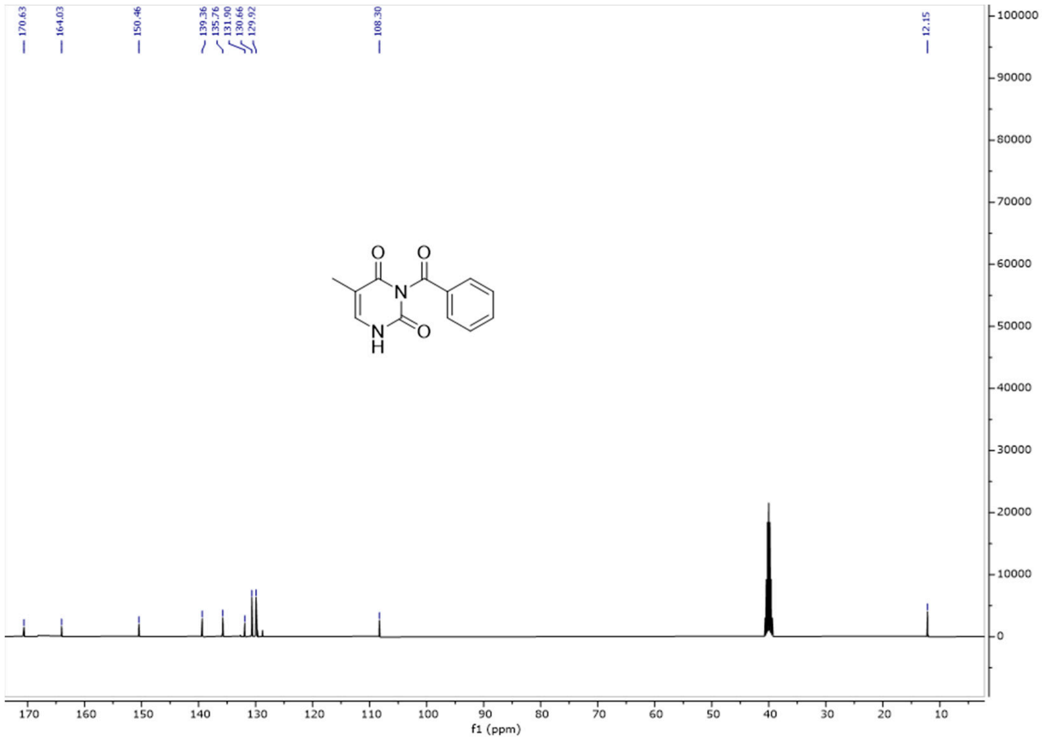

Figure S9: ^{13}C NMR of compound 9 (d6-DMSO)

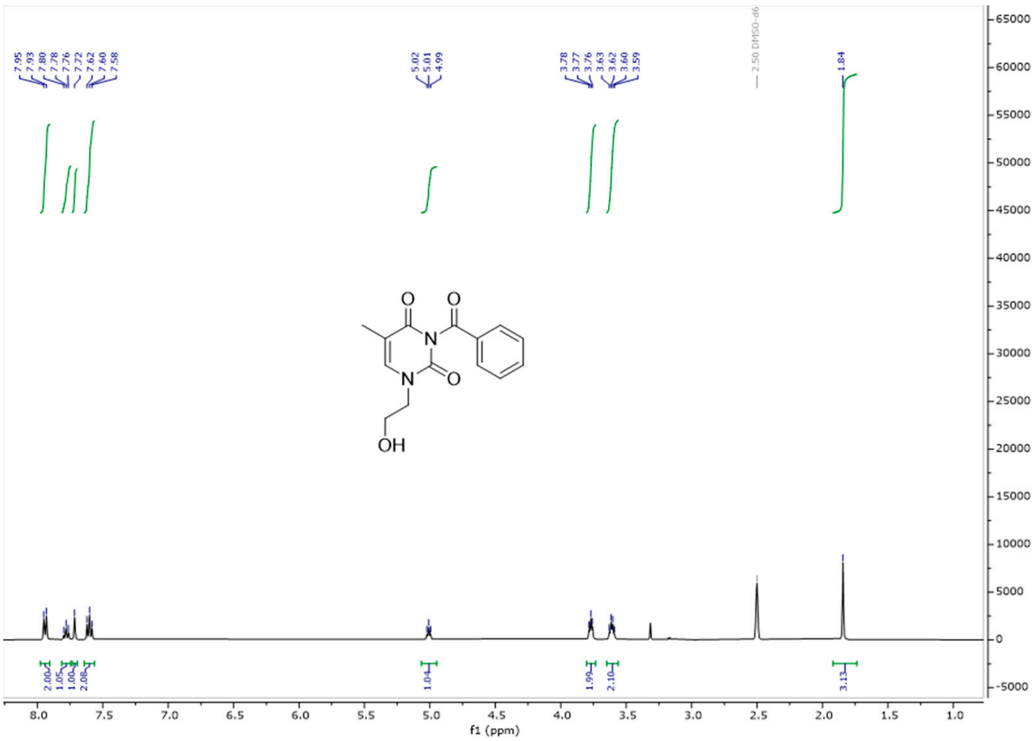

Figure S10: ^{1}H NMR of compound 10 (d6-DMSO)

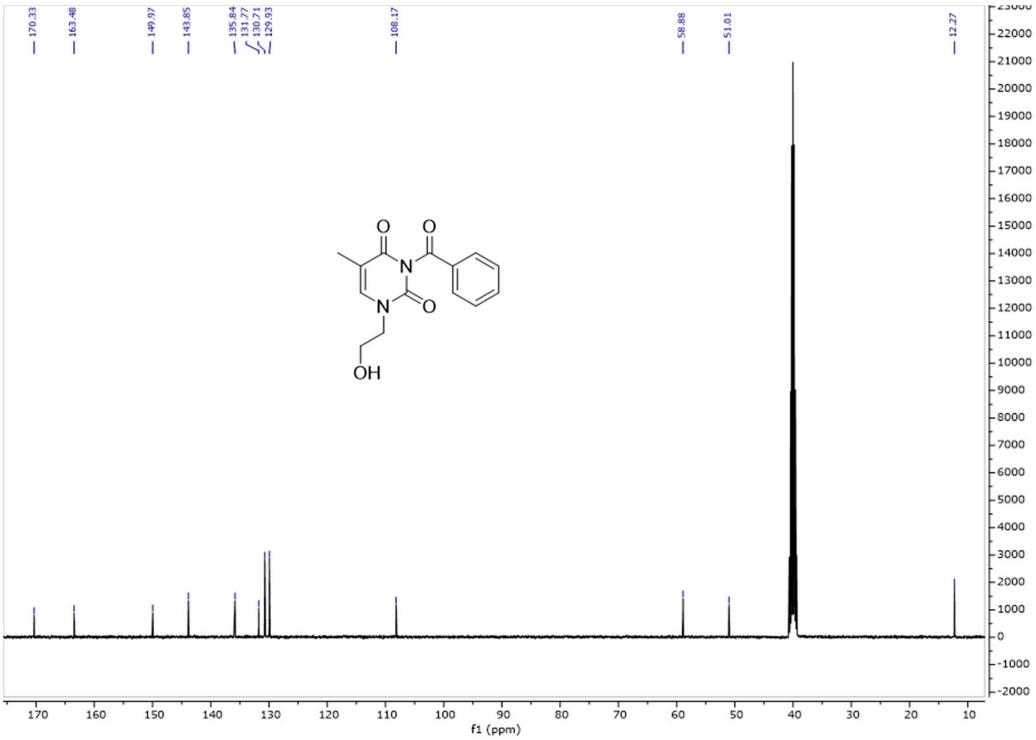

Figure S11: ^{13}C NMR of compound 10 (d6-DMSO)

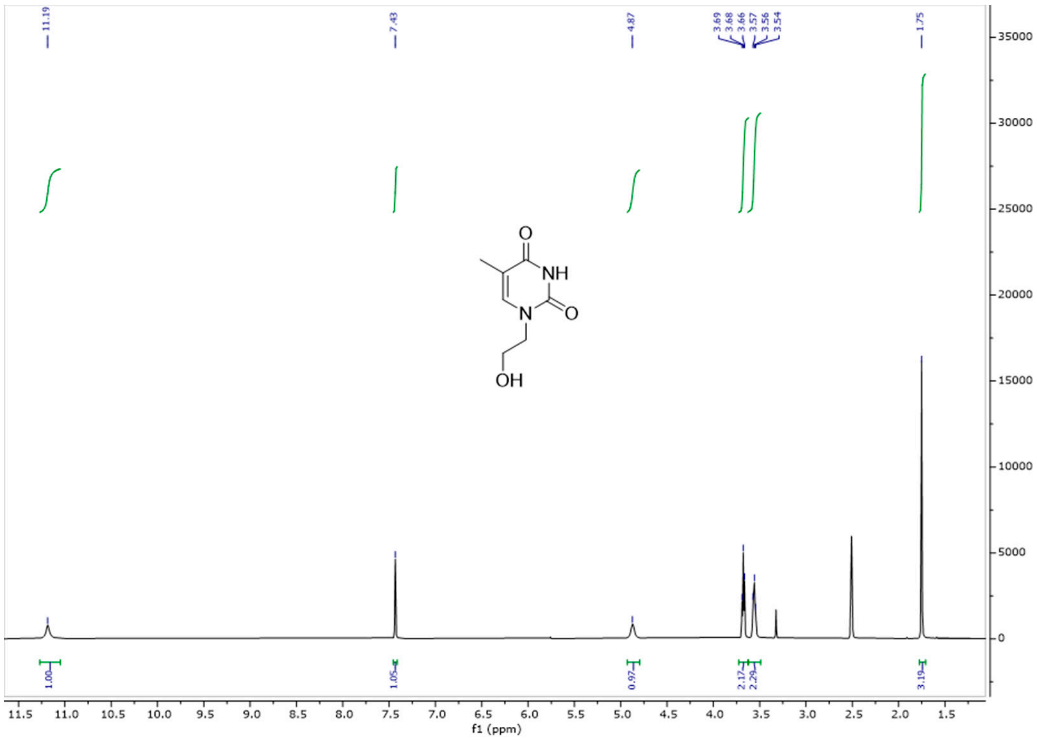

Figure S12: ^{1}H NMR of compound 11 (d6-DMSO)

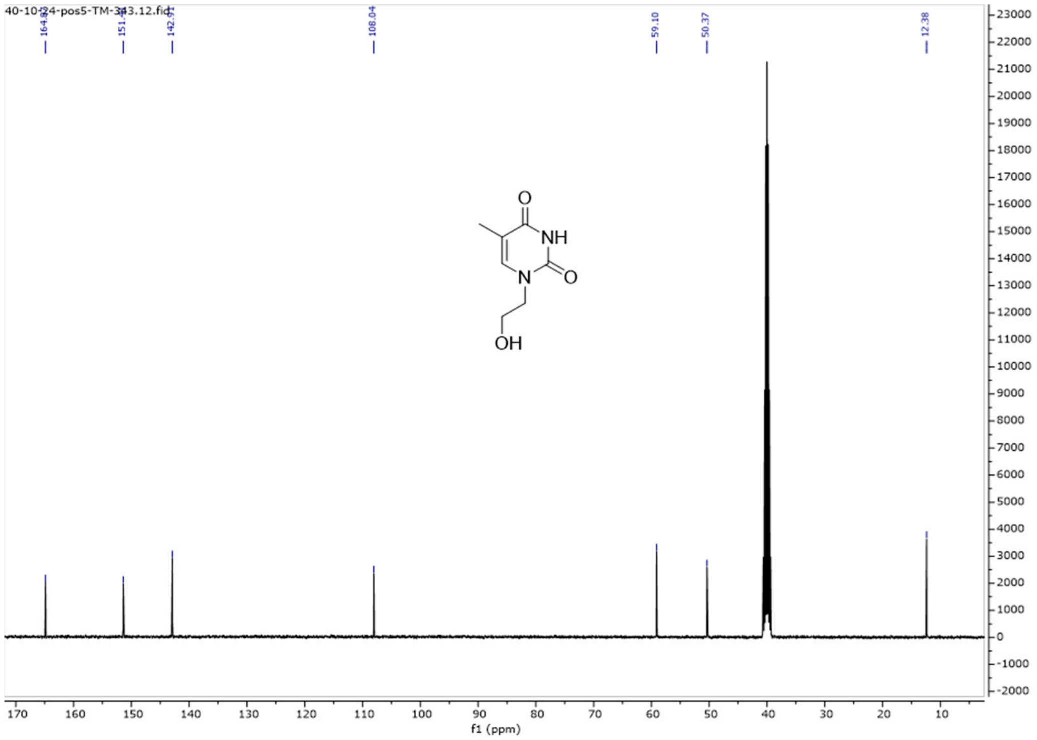

Figure S13: ^{13}C NMR of compound 11 (d6-DMSO)
